# Association between inflammatory biomarkers and cataracts: validation from the NHANES database and clinical studies

**DOI:** 10.3389/fnut.2026.1782065

**Published:** 2026-04-28

**Authors:** Jingjing Ding, Ning Bao, Shengzhen Liu, Liming Tao, Jinsong Zhang

**Affiliations:** 1 Department of Ophthalmology, Anqing Second People's Hospital, Anqing, China; 2 Department of Ophthalmology, The Second Affiliated Hospital of Anhui Medical University, Hefei, China

**Keywords:** cataracts, clinical studies, inflammatory and nutritional biomarkers, NHANES database, population-based study

## Abstract

**Introduction:**

Cataract-associated blindness has increasingly emerged as a major public health issue. Although inflammatory and nutritional status are well-established contributors to various systemic diseases, their associations with cataracts remain underexplored. This study leveraged the NHANES database to investigate the associations between inflammatory and nutritional biomarkers and cataract surgery history prevalence, with subsequent validation using hospital-based clinical data.

**Methods:**

Participants with complete data from the 2005–2008 NHANES cycles were included. Baseline characteristics were first compared between groups. Multivariate logistic regression and restricted cubic spline (RCS) analyses were then employed to assess the associations between inflammation-nutrition indices and cataract surgery history prevalence. Subgroup analyses and interaction tests were conducted to evaluate consistency across diverse populations. Receiver operating characteristic (ROC) curve analysis determined the predictive utility for cataract surgery history prevalence. Sensitivity analyses were performed to bolster the robustness of the results. Finally, findings were validated using clinical cohort data.

**Results:**

The study included 8,194 participants. After adjustment for pertinent confounders, log-transformed AISI (Ln-AISI), NAR (Ln-NAR), and MAR (Ln-MAR) exhibited a linear positive association with cataract surgery history prevalence. Subgroup analyses demonstrated no significant interactions in strata defined by age, gender, smoking status, alcohol consumption, hypertension, diabetes, and body mass index (all *p*-values for interaction > 0.05). Two sensitivity analyses confirmed the robustness of these associations. ROC analysis revealed that Ln-MAR (AUC = 0.592) showed the strongest association with cataract surgery history prevalence among the three markers, although the discriminatory ability of all three indices remained modest. In the clinical cohort, the cataract group displayed elevated MAR and NAR levels but reduced ALI levels compared to the non-cataract group.

**Conclusion:**

This study demonstrates positive correlations between MAR, NAR, and AISI with cataract surgery history prevalence, underscoring the intimate link between systemic inflammation, nutritional status, and cataract pathogenesis. Strategies aimed at mitigating systemic inflammation and optimizing nutritional balance may hold promise for cataract prevention.

## 1 Introduction

Cataracts represent one of the foremost causes of visual impairment worldwide, affecting over 15 million individuals globally, with a disproportionate impact on the elderly population (1, 2). Characterized by lens opacification, the condition advances via multifaceted mechanisms, including oxidative stress, metabolic dysregulation, and chronic low-grade inflammation (3, 4). Emerging evidence underscores the critical interplay between systemic inflammation and nutritional status in the pathogenesis of ocular disorders. Disruptions in this equilibrium may expedite lens degeneration through pathways such as endothelial dysfunction and lens protein aggregation (5, 6).

Composite indices derived from routine blood parameters have attracted considerable attention as readily accessible biomarkers for evaluating the interplay between inflammation and nutritional status. These indices include the monocyte-to-albumin ratio (MAR), which reflects the interaction between inflammatory status and nutritional condition (7); the neutrophil-to-albumin ratio (NAR), which reflects neutrophil-predominant inflammation normalized to serum albumin concentrations (8); the advanced lung cancer inflammation index (ALI), originally developed for malignancies yet applicable to broader inflammatory conditions (9); the prognostic nutritional index (PNI), which integrates albumin and lymphocyte metrics to assess nutritional risk (10); the Systemic Inflammatory Aggregate Index (AISI), which amalgamates neutrophils, monocytes, platelets, and lymphocytes to provide a comprehensive inflammatory score (11); and the Hemoglobin-Albumin-Lymphocyte-Platelet (HALP) score, which incorporates hemoglobin to quantify the inflammatory burden associated with anemia (12).

The National Health and Nutrition Examination Survey (NHANES) is a nationally representative, cross-sectional survey program designed to collect detailed clinical, biochemical, and nutritional data from the general population. Prior investigations leveraging NHANES data have elucidated associations between dietary inflammatory patterns, systemic inflammation levels, and ocular health, indicating that pro-inflammatory states may elevate the risk of cataract development (6, 13). Nonetheless, research examining the integrated role of serum-derived inflammation-nutrition biomarkers in cataract pathogenesis remains sparse. This lacuna is noteworthy, as these markers are cost-effective, readily derived from routine complete blood counts and biochemical assays, and offer substantial promise for early risk stratification. The present analysis, drawing upon NHANES data, investigates the associations between the monocyte-to-albumin ratio (MAR), NAR, ALI, PNI, AISI, and HALP score with cataract prevalence, providing preliminary epidemiological evidence for subsequent cataract-related research.

## 2 Methods

### 2.1 Study design and data sources

This study draws upon data from NHANES to investigate the associations between inflammation- and nutrition-related biomarkers and cataract surgery history. Validation was performed using a clinical cohort from Anqing Second People’s Hospital. The NHANES is administered by the National Center for Health Statistics (NCHS) under the CDC. The publicly accessible data have received approval from the NCHS Institutional Review Board, with informed consent obtained from all participants. Data from the 2005–2008 cycles were selected for analysis, as these cycles included cataract surgery -related information for adults aged 20 years and older.

**Figure 1:**
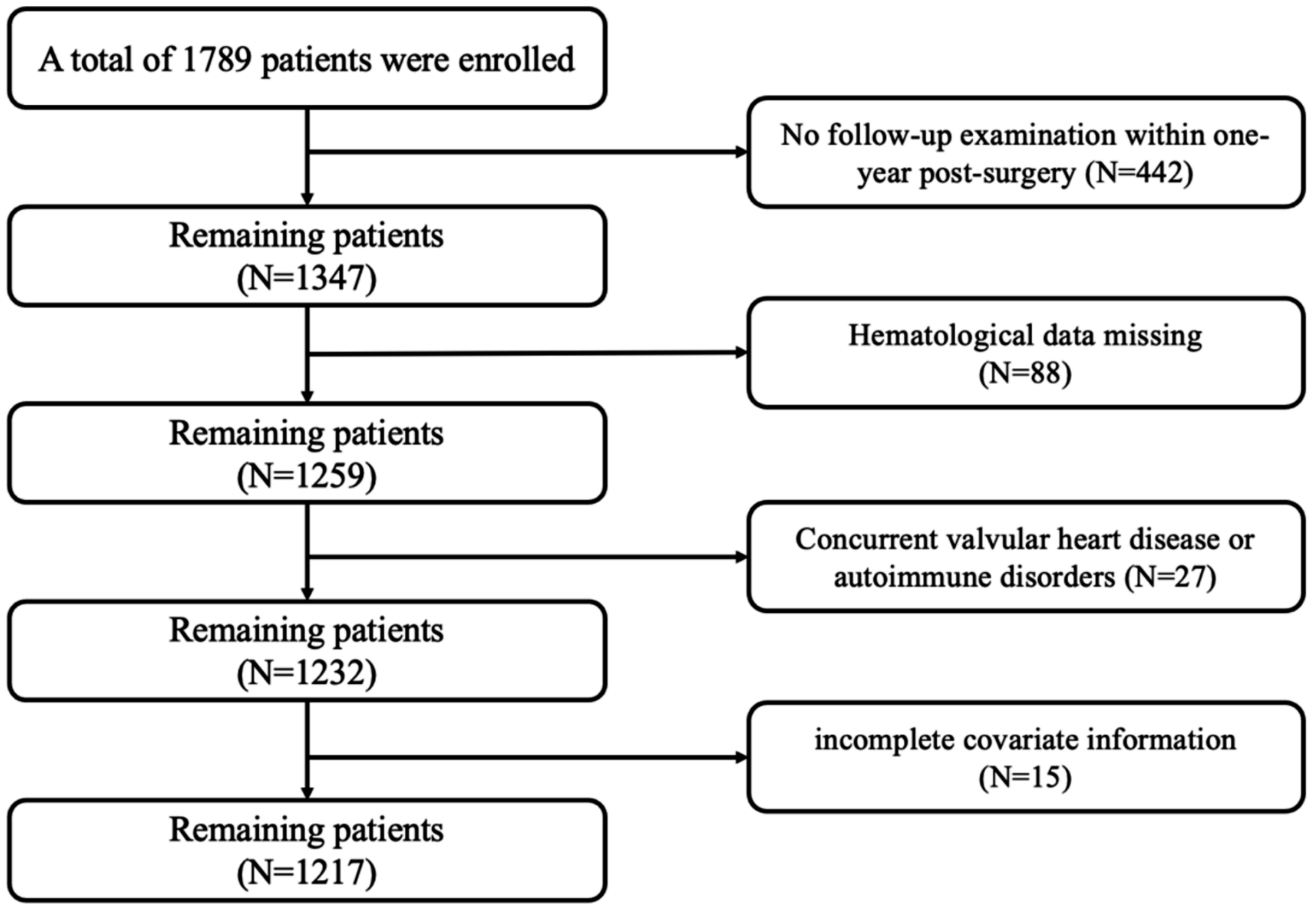
Flowchart of participants for the NHANES 2005–2008.

A total of 20,497 participants were extracted from the NHANES 2005–2008 cycles to form the study population. Participants were excluded if they had missing data for any of the following independent variables: MAR, NAR, PNI, AISI, ALI or HALP; if they had missing cataract surgery history information; or if they had missing key covariates. Ultimately, 8,194 participants were included. Detailed information is shown in Figure 1.

### 2.2 Definition of independent variables (inflammation and nutrition markers)

**Figure 2:**
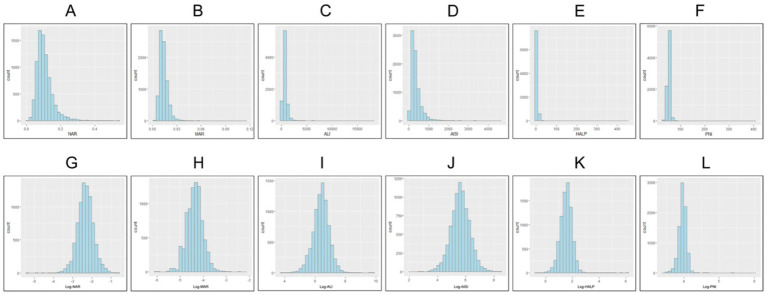
The distribution of inflammatory and nutritional markers. Panels **(A–F)** present the frequency distribution of the original levels of inflammatory and nutritional markers: **(A)** NAR; **(B)** MAR; **(C)** ALI; **(D)** AISI; **(E)** HALP; **(F)** PNI. Panels **(G–L)** display the frequency distribution of log-transformed levels of the corresponding markers: **(G)** Log-NAR; **(H)** Log-MAR; **(I)** Log-ALI; **(J)** Log-AISI; **(K)** Log-HALP; **(L)** Log-PNI.

Independent variables include the following biomarkers calculated from complete blood count and biochemical indicators, measured using automated instruments (Beckman Coulter systems) at NHANES Mobile Examination Centers (MECs):

MAR: Monocyte count/Albumin level (g/dL).
NAR: Neutrophil count/Albumin level (g/dL).
PNI: 10 × Albumin level (g/dL) + 0.005 × Lymphocyte count (/μL).
AISI: (Neutrophil × Monocyte × Platelet count)/Lymphocyte count.
ALI: Body Mass Index (BMI) × Albumin level (g/dL)/Neutrophil-Lymphocyte Ratio (NLR).
HALP: (Hemoglobin level × Albumin level × Lymphocyte count)/Platelet count. All continuous biomarkers were log-transformed to achieve normality for subsequent linear regression analysis, and the log-transformed variables (Ln-AISI, Ln-NAR, Ln-MAR, Ln-ALI, Ln-HALP, Ln-PNI) were used in all multivariate regression models. See Figure 2.

### 2.3 Definition of cataract surgery history

In the absence of direct lens examination in NHANES, the outcome indicator in this study was defined as self-reported cataract surgery history rather than clinically diagnosed cataract. Participants who answered “yes” to the question “Have you ever had a cataract surgery?” (VIQ071) were considered to have a cataract surgery history. This method has been widely adopted in previous studies and is highly reliable and representative (14, 15).

### 2.4 Covariates

The following covariates were selected based on the literature and potential confounders. They were obtained through self-reported questionnaires, physical examinations, and laboratory tests. Demographic characteristics: Age (continuous variable); gender (male/female); race (non-Hispanic White, non-Hispanic Black, Hispanic/Latino, etc.); education level (below high school, high school and above); and economic status (household income poverty ratio <1.0; ≥1.0). Lifestyle habits: Unhealthy habits include smoking (never smoked/former smoker/current smoker) and alcohol consumption (yes or no, defined as having consumed alcohol on ≥12 occasions in one’s lifetime). Complications: Hypertension (physician diagnosis, use of antihypertensive medication, systolic blood pressure ≥140 mmHg or diastolic blood pressure ≥90 mmHg); diabetes (physician diagnosis, use of antidiabetic medication or insulin, HbA1c ≥ 6.5%); cardiovascular complications (self-reported angina, coronary heart disease, congestive heart failure or stroke). Body Mass Index (BMI): Calculated as weight (kg)/height^2^ (m^2^), categorized as <18.5, 18.5–24.9 or ≥25 kg/m^2^.

### 2.5 Statistical analysis

This study took the complex sampling design of NHANES into account by applying the correct sampling weights and incorporating the clustering and stratification variables in the analyses. Continuous variables are expressed as mean ± standard deviation (SD), while categorical variables are presented as unweighted counts and weighted percentages. Group differences were evaluated using t-tests for continuous variables and χ^2^ tests for categorical variables. To examine the associations between the independent variables and cataract surgery history prevalence, we constructed multivariate logistic regression models: Model 1: Unadjusted. Model 2 age, sex, and race. Model 3 was further adjusted for education, socioeconomic status, lifestyle habits (smoking and alcohol consumption), comorbidities (hypertension, diabetes, and cardiovascular complications), and BMI. Restricted cubic spline (RCS) models were used to explore non-linear relationships, and subgroup analyses were conducted to assess interactions. Odds ratios (OR) and 95% confidence intervals (CI) were reported to quantify the strength of the association, and all OR estimates were derived from log-transformed continuous biomarker variables. All analyses were performed using R software (version 4.2.0), with a two-sided *p* < 0.05 being considered statistically significant.

### 2.6 Clinical validation cohort

A single-center retrospective clinical cohort study was conducted to validate the findings of the NHANES analysis, with all participants recruited from the Department of Ophthalmology, Anqing Second People’s Hospital, from January 2023 to December 2024.

1. Inclusion criteria: (1) Aged ≥18 years; (2) Complete clinical and laboratory examination data (including complete blood count, serum albumin, and anthropometric indicators); (3) Clear diagnosis of cataract or non-cataract confirmed by ophthalmic examination.
2. Exclusion criteria: (1) Ocular diseases other than cataract (glaucoma, age-related macular degeneration, diabetic retinopathy); (2) Severe systemic diseases (malignant tumors, severe liver and kidney failure, autoimmune diseases); (3) Missing key laboratory or clinical data.
3. Cataract diagnostic method: All participants underwent slit-lamp biomicroscopy by an ophthalmologist who was blinded to the laboratory data. Cataract was diagnosed if lens opacification was observed in one or both eyes, and non-cataract was defined as no lens opacification and normal ocular examination.
4. Laboratory measurements: The detection of complete blood count and serum albumin was consistent with the NHANES cohort, using an automated machine to ensure the uniformity of biomarker measurement methods. MAR, NAR, AISI, and ALI were calculated using the same formulas as the NHANES cohort.
5. Statistical methods: Due to the relatively small sample size (*n* = 227) of the clinical validation cohort, only descriptive statistical analysis and group comparison were performed. Continuous variables were compared using an independent samples t-test, and categorical variables using the χ^2^ test. Multivariate logistic regression or confounder adjustment was conducted, and the results are only used for preliminary validation of the association trend of biomarkers in the clinical population.

## 3 Results

### 3.1 Baseline characteristics

Table 1 shows the baseline characteristics of participants. A total of 8,194 eligible participants were enrolled, with a cataract surgery history of 8.9%. The mean age was 46.45 (16.49), and 51.7% were female. Significant differences were observed between the cataract surgery history and non-cataract surgery history groups regarding age, gender, ethnicity, education, socioeconomic status, marital status, unhealthy lifestyle habits, and comorbidities. Regarding inflammatory and nutritional markers, participants with a history of cataract surgery exhibited higher MAR, NAR, and AISI levels, but lower ALI and PNI levels.

**Table 1:** Characteristics of participants stratified by cataract surgery history from NHANES 2005–2008.

Variables	All	Non-cataract surgery history	Cataract surgery history	*p*-value
Number		7461	733	
Gender (*N*, %)				<0.001
Male	4004 (48.3)	3666 (49.0)	338 (38.3)	
Female	4190 (51.7)	3795 (51.0)	395 (61.7)	
Age [years, mean (SD)]	46.45 (16.49)	44.65 (15.23)	72.09 (11.59)	<0.001
Ethnicity (*N*, %)				<0.001
Mexican American	1509 (7.9)	1454 (8.3)	55 (2.6)	
Other Hispanic	573 (3.9)	535 (4.0)	38 (2.3)	
Non-Hispanic White	4146 (72.7)	3619 (71.8)	527 (85.9)	
Non-Hispanic Black	1663 (10.2)	1566 (10.6)	97 (5.8)	
Other	303 (5.1)	287 (5.3)	16 (3.4)	
Education (*N*, %)				<0.001
Less than 9th grade	941 (5.8)	791 (5.3)	150 (13.2)	
9-11th grade	1341 (12.1)	1222 (11.9)	119 (14.5)	
High school graduate or equivalent	1973 (24.6)	1784 (24.3)	189 (29.1)	
Some colleges or AA degree	2257 (30.7)	2097 (31.1)	160 (25.1)	
College graduate or above	1682 (26.8)	1567 (27.4)	115 (18.1)	
BMI (*N*, %)				0.152
<25 kg/m ^2^	2414 (32.3)	2191 (32.2)	223 (32.9)	
25–30 kg/m ^2^	2847 (33.9)	2573 (33.7)	274 (37.0)	
>30 kg/m ^2^	2933 (33.8)	2697 (34.1)	236 (30.1)	
Economic level (*N*, %)				<0.001
<1	1514 (11.9)	1415 (12.2)	99 (8.2)	
1–3	3449 (35.4)	3012 (33.8)	437 (58.5)	
>3	3231 (52.7)	3034 (54.1)	197 (33.3)	
Marital status (*N*, %)				<0.001
Married or living with a partner	5137 (66.0)	4745 (66.8)	392 (54.9)	
Unmarried or other	3,057 (34.0)	2716 (33.2)	341 (45.1)	
Alcohol consumption (*N*, %)				<0.001
Yes	5748 (74.8)	5309 (75.8)	439 (61.0)	
No	2446 (25.2)	2152 (24.2)	294 (39.0)	
Smoking status (*N*, %)				<0.001
Never	4266 (51.8)	3923 (52.1)	343 (47.1)	
Now	1822 (23.2)	1752 (24.2)	70 (9.5)	
Former	2106 (25.0)	1786 (23.7)	320 (43.3)	
Hypertension (*N*, %)				<0.001
Yes	2734 (29.9)	2296 (27.8)	438 (58.8)	
No	5460 (70.1)	5165 (72.2)	295 (41.2)	
Diabetes (*N*, %)				<0.001
Yes	1320 (11.8)	1073 (10.5)	247 (30.1)	
No	6874 (88.2)	6388 (89.5)	486 (69.9)	
CHD (*N*, %)				<0.001
Yes	316 (3.1)	233 (2.5)	83 (11.2)	
No	7878 (96.9)	7228 (97.5)	650 (88.8)	
CHF (*N*, %)				<0.001
Yes	246 (2.1)	170 (1.5)	76 (10.1)	
No	7948 (97.9)	7291 (98.5)	657 (89.9)	
Angina (*N*, %)				<0.001
Yes	219 (2.1)	163 (1.8)	56 (7.1)	
No	7975 (97.9)	7298 (98.2)	677 (92.9)	
Stroke (*N*, %)				<0.001
Yes	295 (2.7)	205 (2.0)	90 (12.7)	
No	7899 (97.3)	7256 (98.0)	643 (87.3)	
ALI	668.84 (430.55)	673.33 (400.91)	605.20 (728.88)	0.001
MAR	0.01 (0.00)	0.01 (0.00)	0.02 (0.01)	<0.001
NAR	0.10 (0.04)	0.10 (0.04)	0.11 (0.04)	0.011
AISI	344.82 (256.83)	339.61 (248.29)	418.64 (348.59)	<0.001
HALP	5.17 (4.75)	5.17 (3.74)	5.20 (11.99)	0.820
PNI	53.29 (7.11)	53.38 (6.31)	52.11 (14.24)	<0.001

SD, standard deviation; BMI, body mass index; CHD, coronary heart disease.

### 3.2 Association between inflammatory and nutritional markers and cataract surgery history prevalence

To investigate the relationship between these factors, three multivariate logistic regression models were constructed (see Table 2). Continuous variable analysis of log-transformed biomarkers revealed significant positive correlations for Ln-NAR, Ln-MAR, and Ln-AISI with cataract surgery history prevalence in the fully adjusted Model 3, with the exact OR (95%CI) of 1.70 (1.05,2.77), 1.56 (1.07,2.26), and 1.32 (1.04,1.66), respectively. This indicates that for each unit increase in the log-transformed NAR, MAR, and AISI, the odds of having a cataract surgery history increased by 70, 56, and 32%, respectively. We then converted these indicators into categorical variables to further examine the relationship between inflammatory and nutritional indicators and cataract surgery history prevalence.

**Table 2:** The relationship between inflammation and nutrition indicators and prevalence of cataract surgery history.

Variable	Model 1		Model 2		Model 3	
	OR (95%CI)	*p-*value	OR (95%CI)	*p-*value	OR (95%CI)	*p-*value
Ln-ALI (continuous)	0.47 (0.39, 0.58)	0.0029	0.79 (0.60, 1.04)	0.0852	0.78 (0.58, 1.04)	0.0818
Ln-ALI (multi-category)						
Q1	ref	ref	ref	ref	ref	ref
Q2	0.72 (0.58, 0.90)	0.0051	1.11 (0.83, 1.50)	0.4650	1.08 (0.76, 1.54)	0.6279
Q3	0.43 (0.33, 0.56)	<0.0001	0.71 (0.52, 0.97)	0.0337	**0.92 (0.48, 1.00)**	**0.0487**
Q4	0.39 (0.29, 0.54)	<0.0001	0.73 (0.47, 1.13)	0.1534	0.70 (0.43, 1.14)	0.1262
The relationship between MAR and incidence of cataract						
Ln-MAR (continuous)	2.85 (2.31, 3.51)	<0.0001	1.62 (1.15, 2.29)	0.0082	**1.56 (1.07, 2.26)**	**0.0245**
Ln-MAR (multi-category)						
Q1	ref	ref	ref	ref	ref	ref
Q2	1.32 (0.93, 1.87)	0.1126	1.26 (0.77, 2.05)	0.3490	1.29 (0.73, 2.27)	0.3235
Q3	1.68 (1.18, 2.38)	0.0055	1.38 (0.92, 2.08)	0.1165	1.41 (0.87, 2.26)	0.1331
Q4	2.40 (1.82, 3.17)	<0.0001	1.49 (1.00, 2.23)	0.0506	1.46 (0.92, 2.31)	0.0910
The relationship between NAR and incidence of cataract						
Ln-NAR (continuous)	1.55 (1.19, 2.01)	0.0019	1.82 (1.16, 2.83)	0.0107	**1.70 (1.05, 2.77)**	**0.0350**
Ln-NAR (multi-category)						
Q1	ref	ref	ref	ref	ref	ref
Q2	1.50 (1.14, 1.97)	0.0054	1.32 (0.92, 1.87)	0.1212	1.29 (0.85, 1.95)	0.1957
Q3	1.31 (0.99, 1.73)	0.0617	1.14 (0.76, 1.70)	0.5099	1.07 (0.67, 1.72)	0.7458
Q4	1.68 (1.21, 2.32)	0.0028	1.86 (1.19, 2.91)	0.0088	1.75 (1.04, 2.95)	0.0373
The relationship between AISI and incidence of cataract						
Ln-AISI (continuous)	1.56 (1.32, 1.83)	<0.0001	1.34 (1.07, 1.67)	0.0123	**1.32 (1.04, 1.66)**	**0.0254**
Ln-AISI (multi-category)						
Q1	ref	ref	ref	ref	ref	ref
Q2	1.15 (0.83, 1.60)	0.3883	1.22 (0.84, 1.77)	0.2889	1.25 (0.81, 1.93)	0.2703
Q3	1.36 (0.99, 1.88)	0.0600	1.30 (0.90, 1.89)	0.1568	1.30 (0.85, 1.98)	0.1888
Q4	1.94 (1.43, 2.62)	0.0001	1.56 (1.06, 2.30)	0.0269	1.53 (0.99, 2.35)	0.0541
The relationship between HALP and incidence of cataract						
Ln-HALP (continuous)	0.56 (0.44, 0.70)	<0.0001	0.91 (0.70, 1.18)	0.4629	0.90 (0.67, 1.21)	0.4564
Ln-HALP (multi-category)						
Q1	ref	ref	ref	ref	ref	ref
Q2	0.69 (0.54, 0.88)	0.0042	0.86 (0.59, 1.26)	0.4319	0.90 (0.59, 1.36)	0.5602
Q3	0.48 (0.38, 0.60)	<0.0001	0.75 (0.54, 1.03)	0.0746	0.78 (0.54, 1.14)	0.1628
Q4	0.53 (0.42, 0.67)	<0.0001	0.87 (0.62, 1.24)	0.4309	0.85 (0.57, 1.27)	0.3705
The relationship between PNI and incidence of cataract						
Ln-PNI (continuous)	0.03 (0.01, 0.09)	<0.0001	0.88 (0.33, 2.33)	0.7884	0.95 (0.32, 2.82)	0.9223
Ln-PNI (multi-category)						
Q1	ref	ref	ref	ref	ref	ref
Q2	0.54 (0.41, 0.70)	<0.0001	0.80 (0.54, 1.18)	0.2453	0.82 (0.52, 1.31)	0.3488
Q3	0.40 (0.30, 0.53)	<0.0001	0.75 (0.51, 1.11)	0.1444	0.76 (0.48, 1.20)	0.1960
Q4	0.31 (0.25, 0.38)	<0.0001	0.93 (0.67, 1.28)	0.6289	0.94 (0.64, 1.37)	0.6908

Model 1: unadjusted.

Model 2: Model 1 + gender, age and race.

Model 3: Model 2 + educational level, CHD, CHF, stroke, angina, economic level, BMI, drink, smoking status, hypertension, and diabetes.

BMI, body mass index; OR, odds ratio; CI, confidence interval; MAR, monocyte-to-albumin ratio; NAR, neutrophil-to-albumin ratio; ALI, advanced lung cancer inflammation index; PNI, prognostic nutritional index; AISI, systemic inflammatory aggregate Index; HALP, hemoglobin-albumin-lymphocyte-platelet.

**Figure 3:**
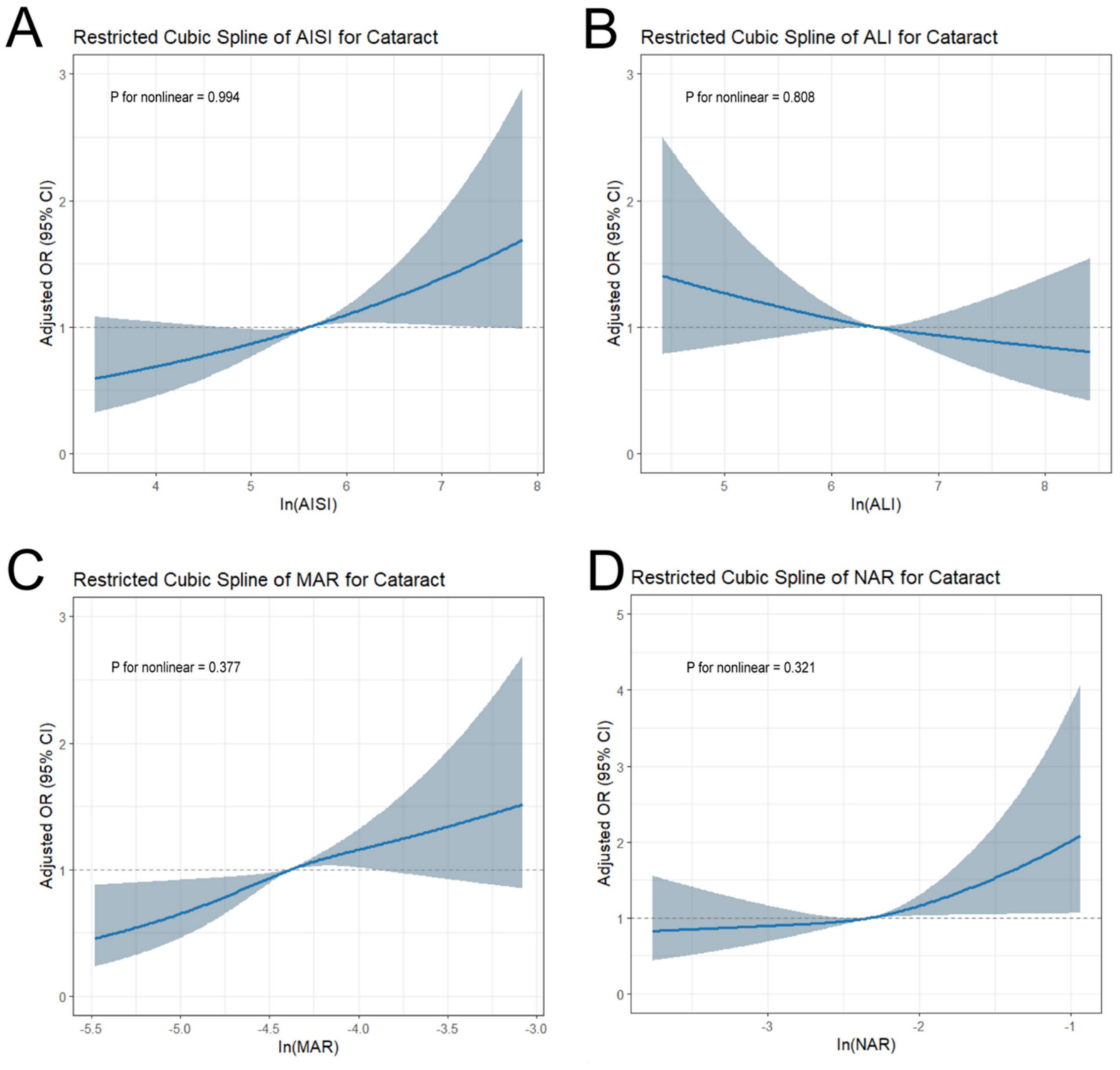
Restricted cubic spline analysis of inflammatory–nutritional indices and cataracts.Panels show the adjusted odds ratios (OR) with 95% confidence intervals (CI) for cataract across the continuous levels of log-transformed indices: **(A)** AISI; **(B)** ALI; **(C)** MAR; **(D)** NAR.

Figure 3 shows the results of the RCS curves, indicating that log-MAR, log-NAR, log-AISI, and log-ALI are linearly correlated with the history of cataract surgery prevalence (*p* for nonlinearity >0.05 for all).

### 3.3 Subgroup analysis

**Figure 4:**
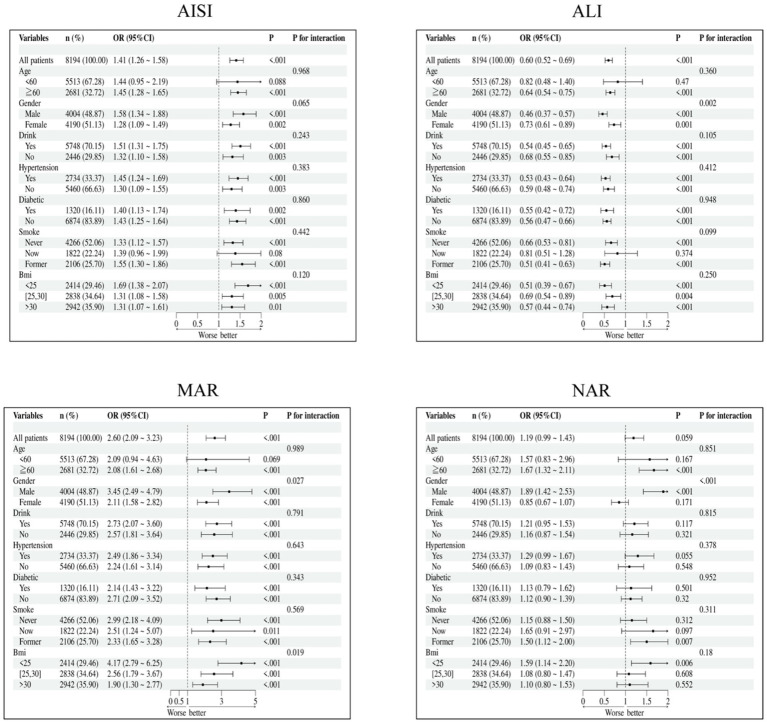
Subgroup analysis of the association between inflammatory–nutritional indices and cataracts.

We conducted subgroup analyses based on age, gender, smoking status, alcohol consumption, hypertension, diabetes, and BMI to investigate whether the relationship between Ln-MAR, Ln-NAR, Ln-AISI, and Ln-ALI and cataract surgery history prevalence remained consistent across subgroups. The association remained consistent across all subgroups, with no statistically significant interaction effects detected (Figure 4).

### 3.4 ROC analysis

**Figure 5:**
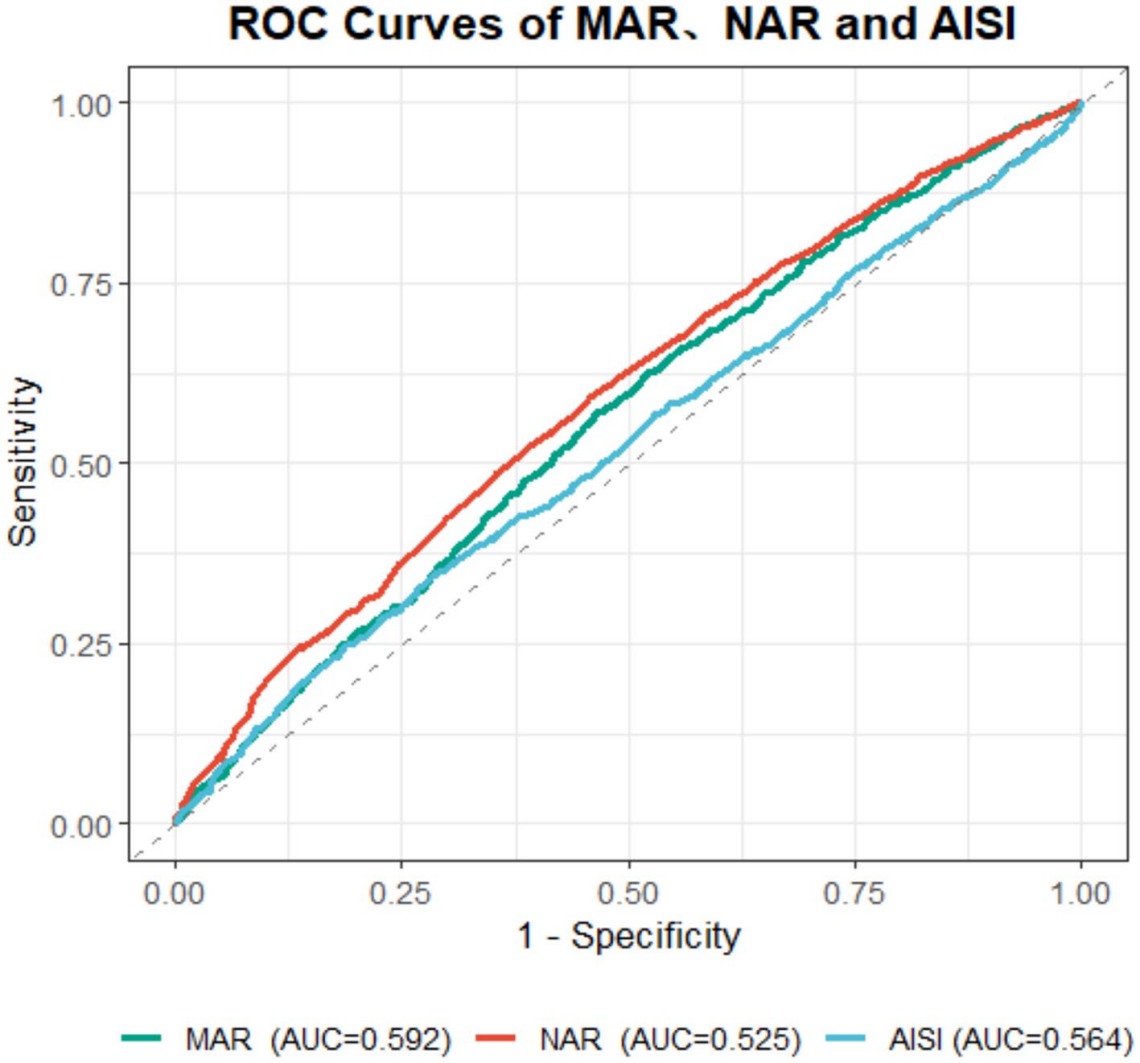
ROC curves and AUC comparisons of the predictive performance of NAR, MAR, and AISI for cataracts.

Figure 5 illustrates the ROC curve analysis, showing that among MAR, NAR, and AISI, MAR exhibited the highest predictive performance for cataract surgery history (AUC = 0.592). All three AUC values were close to 0.5, indicating that the three biomarkers have limited predictive ability and low discriminatory accuracy for cataract surgery history, with no clinical diagnostic value. The DeLong test was applied to examine the relationships among these three variables (see Table 3).

**Table 3:** Comparison of AUCs between inflammation and nutrition markers using DeLong’s test.

Disease	Comparison	AUC of marker 1	AUC of marker 2	*Z* statistic (DeLong test)	*p*
Cataract	MAR vs. AISI	0.592	0.564	2.710	0.007
	MAR vs. NAR	0.592	0.525	5.687	<0.001
	AISI vs. NAR	0.564	0.525	4.611	<0.001

This table presents the results of DeLong’s test for comparing AUCs between MAR, NAR, and AISI for identifying cataract. Statistically significant differences (*p* < 0.05) suggest that the composite ratio provides superior diagnostic performance.

### 3.5 Sensitivity analysis

To enhance the reliability of the results, two sensitivity analyses were conducted: a reanalysis using unweighted and multiple-imputed data (Supplementary Figures S1, S2). The results of the multivariate logistic regression analysis confirmed that the relationships between Ln-MAR, Ln-NAR, Ln-AISI, and Ln-ALI and the history of cataract surgery prevalence remained stable.

**Figure 6:**
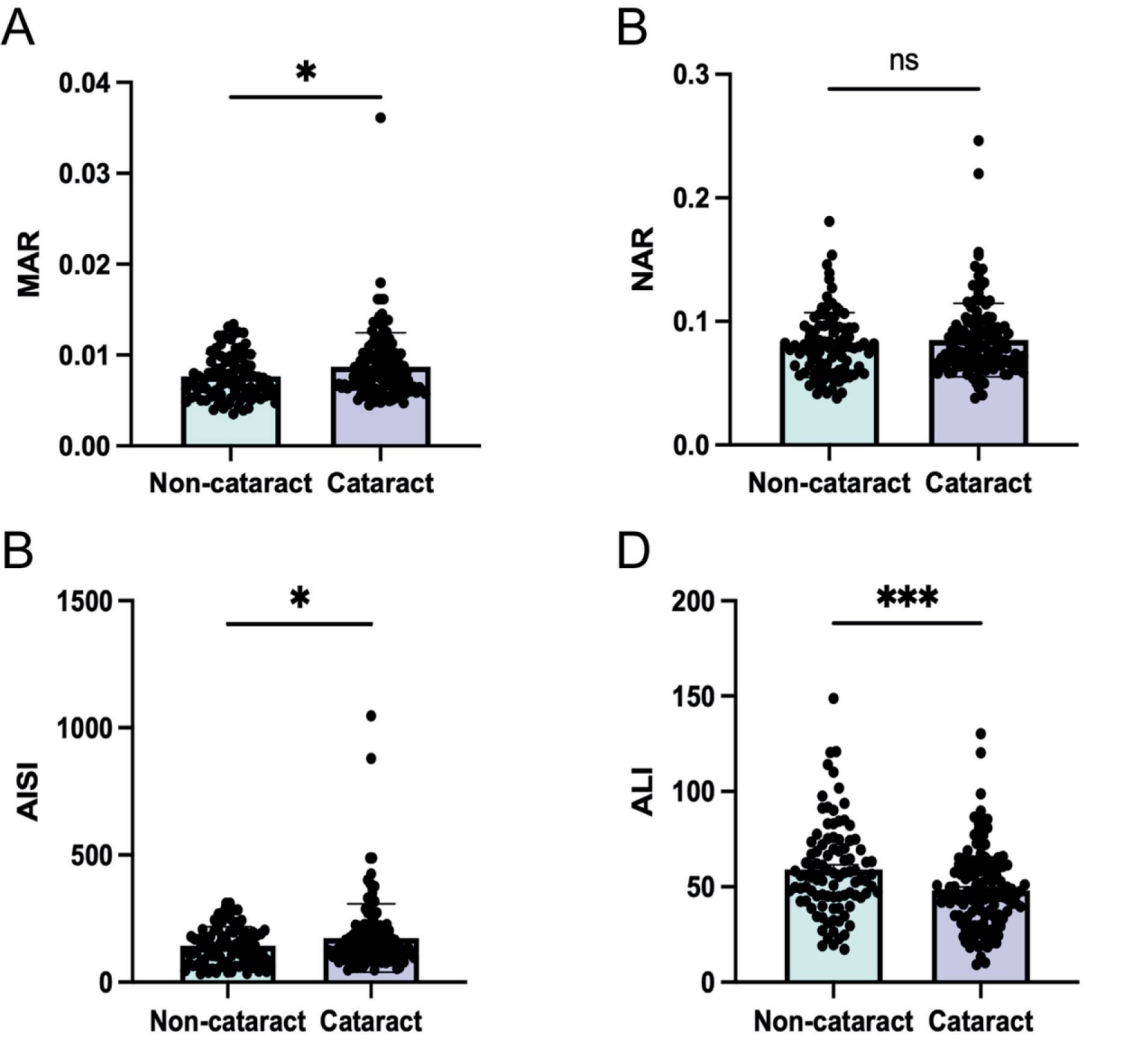
Panels show the distribution of indicator levels in the two groups: **(A)** MAR; **(B)** NAR; **(C)** AISI; **(D)** ALI. ns: not significant; *p < 0.05; ***p < 0.001.

### 3.6 Clinical data validation

In a retrospective cohort study conducted at Anqing Second People’s Hospital, a total of 227 patients were enrolled, comprising 95 individuals with cataracts and 132 without cataracts. Table 4 presents the baseline characteristics of the study population. Comparative analyses of ALI, NAR, MAR, and AISI between the cataract and non-cataract groups revealed a significant increase in MAR and AISI levels, and a significant decrease in ALI levels in the cataract group (Figure 6). After adjusting for covariates, including age, this association with cataract prevalence remained statistically significant. No significant difference was observed in NAR between the two groups (Table 5). These findings suggest that elevated MAR and AISI may serve as risk factors for increased cataract prevalence, while higher ALI appears to exert a protective effect.

**Table 4:** Characteristics of participants stratified by cataract from clinical study.

Variables	All	Non-cataract	Cataract
Number	227	132	95
Gender (*N*, %)			
Male	99 (43.6)	42 (44.2)	57 (43.2)
Female	128 (56.4)	53 (55.8)	75 (56.8)
Age [years, mean (SD)]	61.94 (17.42)	45.31 (12.59)	73.91 (8.04)
BMI (*N*, %)			
<25 kg/m ^2^	158 (69.6)	61 (64.2)	97 (73.5)
25–30 kg/m ^2^	62 (27.3)	30 (31.6)	32 (24.2)
>30 kg/m ^2^	7 (3.1)	4 (4.2)	3 (2.3)
Hypertension (*N*, %)			
Yes	85 (37.4)	13 (13.7)	72 (54.5)
No	142 (62.6)	82 (86.3)	60 (45.5)
Leukocyte	5.86 (1.41)	5.95 (1.44)	5.79 (1.39)
Neutrophil	3.59 (1.12)	3.60 (1.14)	3.59 (1.12)
Monocular	0.36 (0.12)	0.34 (0.11)	0.37 (0.12)
Lymphocyte	1.73 (0.55)	1.85 (0.53)	1.65 (0.56)
Hemoglobin	131.96 (13.71)	137.46 (14.29)	128.01 (11.83)
Platelet count	197.71 (46.52)	201.47 (47.61)	195.01 (45.71)
Albumin	43.61 (3.89)	44.76 (3.84)	42.78 (3.72)
MAR	0.01 (0.00)	0.01(0.00)	0.01(0.00)
NAR	0.08 (0.03)	0.08 (0.03)	0.08 (0.03)
AISI	160.93 (113.96)	143.15 (74.76)	173.73 (134.17)
PNI	52.27 (4.86)	54.00 (4.77)	51.03 (4.54)
ALI	52.66 (22.77)	59.04 (24.36)	48.08 (20.44)
HALP	52.45 (19.60)	58.72 (19.97)	47.93 (18.10)

SD, standard deviation; BMI, body mass index.

**Table 5:** The relationship between inflammation and nutrition indicators and the prevalence of cataract surgery history.

Variables	Model 1		Model 2		Model 3	
	OR (95%CI)	*p-*value	OR (95%CI)	*p-*value	OR (95%CI)	*p-*value
NAR	1.04 (0.82–1.32)	0.317	1.02 (0.79–1.29)	0.284	1.01 (0.77–1.27)	0.362
MAR	1.68 (1.25–2.27)	0.004	1.59 (1.18–2.14)	0.038	1.52 (1.12–2.06)	0.042
AISI	1.83 (1.36–2.47)	<0.001	1.74 (1.29–2.35)	<0.021	1.66 (1.22–3.25)	0.037
ALI	0.62 (0.47–0.81)	0.003	0.67 (0.51–0.88)	0.011	0.71 (0.54–0.93)	0.028

Model 1: unadjusted.

Model 2: Model 1 + gender and age.

Model 3: Model 2 + hypertension and BMI.

BMI, body mass index; OR, odds ratio; CI, confidence interval.

## 4 Discussion

This study, which was based on the NHANES 2005–2008 cohort for which complete information was available, found significant associations between inflammatory-nutritional biomarkers (MAR, NAR, AISI, and ALI) and the history of cataract surgery prevalence among US adults. Validation of the clinical data confirmed that the cataract group exhibited higher MAR and AISI levels, as well as lower ALI scores, than the non-cataract group.

While direct studies on MAR and NAR in relation to cataracts are limited, there is evidence from similar indices, such as the neutrophil-to-albumin ratio (NAR) and systemic immune-inflammation index (SII), that they are positively correlated with ocular diseases (16, 17). This suggests that elevated MAR/NAR levels may indicate a greater inflammatory burden in relation to nutritional status. After adjusting for relevant confounders, the likelihood of a history of cataract surgery prevalence remained elevated. Similarly, higher AISI levels were found to positively correlate with a history of cataract surgery prevalence, while higher ALI levels were found to offer protection by indicating better nutritional reserves under lower inflammation (18).

Notably, the association between the NAR index reached statistical significance in the NHANES population-based analysis but not in the clinical validation cohort, which warrants careful interpretation. NHANES is a nationally representative survey with an extremely large sample size, thereby conferring adequate statistical power. In contrast, our clinical validation cohort is a relatively small, single-center clinical sample. Second, there are substantial differences in baseline characteristics between the general population of NHANES and the selected clinical patients: the former encompasses a wide spectrum of inflammatory and nutritional statuses with high variability in NAR values, whereas the latter consists of homogeneous patients with already elevated baseline inflammatory levels. The restricted range of biomarker values in the clinical population may attenuate the strength of the observed association. Geographical and ethnic heterogeneity with direct cohort evidence: Critically, the NHANES cohort represents a multi-ethnic US population, while our clinical cohort consists of Han Chinese participants recruited from Anhui province. These findings highlight that while NAR shows promise as a population-level screening biomarker, further validation in larger, multi-center clinical cohorts is needed to confirm its utility in specific patient populations.

These associations highlight the interplay between systemic inflammation and nutritional deficiencies in cataract pathogenesis (19, 20). Chronic, low-grade inflammation, as measured by MAR, NAR, and AISI, promotes oxidative stress by activating neutrophils and monocytes, which release reactive oxygen species (ROS) and pro-inflammatory cytokines (IL-1, IL-8, and TNF-*α*). These mediators can disrupt the integrity of lens epithelial cells and induce protein aggregation in the avascular lens, resulting in opacity (21, 22). Albumin, a key component of MAR, NAR and ALI, acts as an antioxidant scavenger, mitigating ROS. Hypoalbuminemia exacerbates damage by reducing buffering capacity (23, 24).

From a nutritional perspective, diets with a high inflammatory potential, such as those with an elevated Dietary Inflammation Index (DII), can exacerbate this process. Pro-inflammatory foods such as saturated fats and refined sugars elevate systemic markers such as C-reactive protein and IL-6, overwhelming the lens’s antioxidant defenses and accelerating protein denaturation (25, 26). Conversely, anti-inflammatory nutrients such as vitamins C and E, lutein and zeaxanthin, and omega-3 fatty acids, which are found in fruits, vegetables, and whole grains, enhance glutathione (GSH) synthesis and ROS neutralization. This nutritional-inflammation interaction may be one of the potential mechanisms underlying the association between the studied biomarkers and cataract, but the causal relationship cannot be confirmed due to the cross-sectional study design. This process can potentially delay cataract development by delivering nutrients via the lens microcirculatory system. Gender-specific effects, such as stronger SII-cataract associations in women, suggest that hormones influence inflammation and necessitate further targeted research rather than clinical interventions (27–29).

Although the lens itself is an avascular tissue, its physiological metabolism and microenvironment are completely dependent on the aqueous humor, which is connected to the systemic circulation (30). Systemic low-grade chronic inflammation can damage the blood-aqueous barrier, allowing circulating pro-inflammatory factors (such as IL-6, TNF-*α*, and hs-CRP) to enter the aqueous humor (31), inducing oxidative stress, apoptosis, and protein denaturation in lens epithelial cells and ultimately driving the occurrence and progression of cataract. Meanwhile, systemic inflammatory response can further aggravate the systemic redox imbalance, which is the core pathological mechanism of cataract formation (32). The nutrients required for the normal metabolism and antioxidant defense of the lens are all derived from the aqueous humor, and the concentration of nutritional components in the aqueous humor is directly determined by their levels in the peripheral blood (33, 34). Systemic nutritional biomarkers (such as albumin, prealbumin, and vitamins) can objectively reflect systemic nutritional status, and their deficiency directly reduces the antioxidant capacity and the ability to maintain protein homeostasis in lens epithelial cells, thereby accelerating lens opacification (35).

Meaningfully, the non-surgical population with early-stage cataract and corresponding visual impairment represents a critical cohort for understanding the early pathological process of cataractogenesis. This group is essential to clarify whether the abnormal changes of biomarkers occur in the early stage of the disease, rather than only emerging in the advanced stage requiring surgical intervention. Future prospective studies focusing on non-surgical early cataract populations, with standardized ophthalmic examinations including slit-lamp lens opacity grading and best-corrected visual acuity assessment, are warranted to further verify the dynamic changes of these biomarkers during cataract progression, and to confirm their clinical value in early risk identification and intervention guidance.

This study has several important limitations that need to be explicitly emphasized. First, the NHANES cohort is a cross-sectional study and cannot establish the temporal sequence between the two or infer a causal relationship. Second, the outcome indicator in the NHANES cohort is defined as self-reported cataract surgery history rather than clinically diagnosed cataract, which may misclassify untreated cataract patients as controls, introduce selection bias, and underestimate the actual prevalence of cataract in the general population. Third, the ROC analysis results show that the studied biomarkers have low AUC values and limited predictive ability for cataract surgery history, with no clinical diagnostic or screening value. Fourth, the clinical validation cohort is a small sample (n = 227) single-center retrospective study, with no multivariate confounder adjustment due to the limited sample size, and the results are only used for preliminary validation of the association trend, with low external generalizability. Fifth, the study has residual confounding bias, as important cataract risk factors such as UV exposure, steroid use, eye trauma, and genetic factors were not included in the covariate adjustment due to the lack of relevant data in the NHANES database and clinical cohort. We were unable to accurately define the specific timing of cataract onset or clarify the precise temporal relationship between disease occurrence and blood biomarker collection.

## 5 Conclusion

In summary, evidence from the NHANES shows that elevated MAR, NAR, and AISI are positively associated with higher cataract surgery prevalence, while ALI provides insights into potential protective associations with inflammation-driven oxidative stress and nutritional regulation. This supports the use of dietary strategies to inhibit cataract formation. Accordingly, more forward-looking prospective and mechanistic studies are warranted in the future to validate and further explore these associations.

## Data Availability

Publicly available datasets were analyzed in this study. This data can be found at: NHANES is a public database, and all researchers can access the data from www.cdc.gov/nchs/nhanes.

## References

[1] Blindness GBD. Vision Impairment C, Vision Loss Expert Group of the Global Burden of Disease S: Causes of blindness and vision impairment in 2020 and trends over 30 years, and prevalence of avoidable blindness in relation to VISION 2020: the right to sight: an analysis for the global burden of disease study. Lancet Glob Health. (2021) 9:e144–60.33275949

[2] Cicinelli MV Buchan JC Nicholson M Varadaraj V Khanna RC. Cataracts. Lancet. (2023) 401:377–89. doi: 10.1016/S0140-6736(22)01839-6, 36565712

[3] Li W Zou Y Zheng W Zhang J Cen C Li R . Transcription factor EGR2 drives cataract formation through IGFBP3-mediated oxidative injury in lens epithelial cells. Free Radic Biol Med. (2025) 241:773–88. doi: 10.1016/j.freeradbiomed.2025.10.009, 41093149

[4] Liu YC Wilkins M Kim T Malyugin B Mehta JS. Cataracts. Lancet. (2017) 390:600–12. doi: 10.1016/S0140-6736(17)30544-5 28242111

[5] Giglio R Milan S Inferrera L Tognetto D D'Esposito F Visalli F . Nutrient-driven antioxidant interventions for prevention of age-related and diabetic cataracts. Nutrients. (2025) 17:1885. doi: 10.3390/nu17111885 40507153 PMC12157995

[6] Wang X Jiang H Zhang C. Association of systemic inflammatory biomarkers with ocular disease: a large population-based cross-sectional study. Eur J Med Res. (2025) 30:206. doi: 10.1186/s40001-025-02473-y, 40140856 PMC11938706

[7] Jin Y Wang X Chen Y Li X Wu X Tian Y . Association of the monocyte-to-albumin ratio with cardiovascular disease and with all-cause and cardiovascular mortality in the general population. Front Cardiovasc Med. (2025) 12:1645793. doi: 10.3389/fcvm.2025.1645793, 41059443 PMC12497811

[8] Chen Y Cao Y Zhu W Huang Z. Exploration of the potential therapeutic benefits of naringenin against diabetic retinopathy through a national comprehensive cross-sectional study and in vitro experiments. Diabetol Metab Syndr. (2025) 17:304. doi: 10.1186/s13098-025-01879-2, 40751167 PMC12315193

[9] Chen L He Y Chen H Cheng J. Combined impact of inflammation, nutrition, and cardiovascular health on cancer survivor mortality: a retrospective NHANES cohort analysis (2005-2018). Expert Rev Anticancer Ther. (2025) 25:1459–69. doi: 10.1080/14737140.2025.2581140, 41146494

[10] Wu Q Ding W You D Ji Y Wang S Jiang D . Prognostic nutritional index, sarcopenia, and risk of mortality: a national population-based study. Nutr Metab (Lond). (2025) 22:106. doi: 10.1186/s12986-025-01005-z, 40999455 PMC12465300

[11] Wang R Chen R Tao W Cheng X. Nonlinear associations between the aggregate index of systemic inflammation and cardiovascular disease in adults: evidence from NHANES 2011-2020. BMC Public Health. (2025) 25:3031. doi: 10.1186/s12889-025-24320-9, 40898106 PMC12406563

[12] He Y Ma Z Chen X Wang J Chen X Deng Z . Association between hemoglobin, albumin, lymphocyte, and platelet score and all-cause and cardiovascular mortality among population with diabetes: evidence from the NHANES 2003-2016. Diabetes Res Clin Pract. (2025) 224:112212. doi: 10.1016/j.diabres.2025.112212, 40345595

[13] Zhang J Xiao L Zhao X Wang P Yang C. Exploring the association between composite dietary antioxidant index and ocular diseases: a cross-sectional study. BMC Public Health. (2025) 25:625. doi: 10.1186/s12889-025-21867-5, 39953504 PMC11829354

[14] Wang CX Fan JW Liu JH Lin SY Hou JJ Jiang ZX . Pan-immune inflammation value: a novel biomarker for cataract. PLoS One. (2025) 20:e0335713. doi: 10.1371/journal.pone.0335713, 41171769 PMC12578218

[15] Wei B Hu X Shu BL Huang QY Chai H Yuan HY . Association of triglyceride-glucose index and derived indices with cataract in middle-aged and elderly Americans: NHANES 2005-2008. Lipids Health Dis. (2025) 24:48. doi: 10.1186/s12944-025-02470-4, 39953544 PMC11827319

[16] He X Dai F Zhang X Pan J. The neutrophil percentage-to-albumin ratio is related to the occurrence of diabetic retinopathy. J Clin Lab Anal. (2022) 36:e24334. doi: 10.1002/jcla.24334, 35285099 PMC8993596

[17] Huang J Wu H Yu F Wu F Hang C Zhang X . Association between systemic immune-inflammation index and cataract among outpatient US adults. Front Med (Lausanne). (2024) 11:1469200. doi: 10.3389/fmed.2024.1469200, 39359932 PMC11445128

[18] Trimarchi G Pizzino F Lilli A De Caterina AR Esposito A Dalmiani S . Advanced lung Cancer inflammation index as predictor of all-cause mortality in ST-elevation myocardial infarction patients undergoing primary percutaneous coronary intervention. J Clin Med. (2024) 13:6059. doi: 10.3390/jcm13206059, 39458009 PMC11508711

[19] Li X Du GL Wu SN Sun YQ Zhang SQ Zhang ZJ . Association between systemic immune inflammation index and cataract incidence from 2005 to 2008. Sci Rep. (2025) 15:499. doi: 10.1038/s41598-024-84204-7, 39747967 PMC11696561

[20] Sutkowy P Lesiewska H Wozniak A Malukiewicz G. Inflammation-involved proteins in blood serum of cataract patients-a preliminary study. Biomedicine. (2023) 11:2607. doi: 10.3390/biomedicines11102607, 37892980 PMC10604040

[21] Liu S Jin Z Xia R Zheng Z Zha Y Wang Q . Protection of human Lens epithelial cells from oxidative stress damage and cell apoptosis by KGF-2 through the Akt/Nrf2/HO-1 pathway. Oxidative Med Cell Longev. (2022) 2022:6933812. doi: 10.1155/2022/6933812, 35222803 PMC8872674

[22] Tjalkens RB Popichak KA Kirkley KA. Inflammatory activation of microglia and astrocytes in manganese neurotoxicity. Adv Neurobiol. (2017) 18:159–81. doi: 10.1007/978-3-319-60189-2_8 28889267 PMC6462217

[23] Soeters PB Wolfe RR Shenkin A. Hypoalbuminemia: pathogenesis and clinical significance. JPEN J Parenter Enteral Nutr. (2019) 43:181–93. doi: 10.1002/jpen.1451, 30288759 PMC7379941

[24] Watanabe K Kinoshita H Okamoto T Sugiura K Kawashima S Kimura T. Antioxidant properties of albumin and diseases related to obstetrics and gynecology. Antioxidants (Basel, Switzerland). (2025) 14:55. doi: 10.3390/antiox14010055, 39857389 PMC11760856

[25] Li LH Lee JC Leung HH Lam WC Fu Z Lo ACY. Lutein supplementation for eye diseases. Nutrients. (2020) 12:1721. doi: 10.3390/nu12061721, 32526861 PMC7352796

[26] Mohammadi S Hosseinikia M Ghaffarian-Bahraman A Clark CCT Davies IG Yousefi Rad E . Dietary inflammatory index and elevated serum C-reactive protein: a systematic review and meta-analysis. Food Sci Nutr. (2023) 11:5786–98. doi: 10.1002/fsn3.3553, 37823095 PMC10563751

[27] Honisch C Rodella U Gatto C Ruzza P Tothova JD. Oxidative stress and antioxidant-based interventional medicine in ophthalmology. Pharm (Basel, Switzerland). (2023) 16:1146. doi: 10.3390/ph16081146, 37631061 PMC10458870

[28] Rasmussen HM Johnson EJ. Nutrients for the aging eye. Clin Interv Aging. (2013) 8:741–8. doi: 10.2147/CIA.S45399, 23818772 PMC3693724

[29] Ruiz-Saavedra S Salazar N Suarez A de Los Reyes-Gavilan CG Gueimonde M Gonzalez S. Comparison of different dietary indices as predictors of inflammation, oxidative stress and intestinal microbiota in middle-aged and elderly subjects. Nutrients. (2020) 12:3828. doi: 10.3390/nu12123828, 33333806 PMC7765160

[30] Schlotzer-Schrehardt U Naumann GO. Ocular and systemic pseudoexfoliation syndrome. Am J Ophthalmol. (2006) 141:921–37. doi: 10.1016/j.ajo.2006.01.047, 16678509

[31] Tu KL Kaye SB Sidaras G Taylor W Shenkin A. Effect of intraocular surgery and ketamine on aqueous and serum cytokines. Mol Vis. (2007) 13:1130–7. 17653058 PMC2779146

[32] Hsueh YJ Chen YN Tsao YT Cheng CM Wu WC Chen HC. The pathomechanism, antioxidant biomarkers, and treatment of oxidative stress-related eye diseases. Int J Mol Sci. (2022) 23:1255. doi: 10.3390/ijms23031255, 35163178 PMC8835903

[33] Glaesser D Iwig M. Increased molar ratio of free fatty acids to albumin in blood as cause and early biomarker for the development of cataracts and Alzheimer's disease. Exp Eye Res. (2024) 243:109888. doi: 10.1016/j.exer.2024.109888, 38583754

[34] Yanshole VV Yanshole LV Snytnikova OA Tsentalovich YP. Quantitative metabolomic analysis of changes in the lens and aqueous humor under development of age-related nuclear cataract. Metabolomics. (2019) 15:29. doi: 10.1007/s11306-019-1495-4, 30830501

[35] Weikel KA Garber C Baburins A Taylor A. Nutritional modulation of cataract. Nutr Rev. (2014) 72:30–47. doi: 10.1111/nure.12077, 24279748 PMC4097885

