# Supplementary material for: Association between inflammatory biomarkers and cataracts: validation from the NHANES database and clinical studies
Source: Front Nutr. 2026 Apr 28;13:1782065. doi: 10.3389/fnut.2026.1782065 (PMC13160836; doi:10.3389/fnut.2026.1782065)
Supplement: Supplementary file 1 [file Table_1.docx]

**Table S1.** Unweighted multivariate logistic regression analysis of inflammatory and nutritional indices and cataract.

| **Variables** | **Model 1** | **Model 2** | **Model 3** |
| --- | --- | --- | --- |
|  | OR (95% CI) *P*-value | OR (95% CI) P-value | OR (95% CI) P-value |
| Ln-NAR | 1.19(0.99, 1.43), 0.059 | 1.44(1.13, 1.84), 0.004 | 1.19(1.02, 1.64), 0.046 |
| Ln-MAR | 2.60(2.10, 3.23), <0.001 | 1.58(1.21, 2.07), <0.001 | 1.50(1.14, 1.98), 0.004 |
| Ln-AISI | 1.41(1.26, 1.58), <0.001 | 1.23(1.07, 1.41), 0.003 | 1.20(1.04, 1.38), 0.010 |
| Ln-ALI | 0.60(0.52, 0.69), <0.001 | 0.95(0.80, 1.12), 0.514 | 0.91(0.76, 1.10), 0.337 |

Model 1: unadjusted

Model 2: Model 1+age, sex and ethnicity

Model 3: Model2 + educational level, marital status, BMI, economic level, smoking status, alcohol consumption, hypertension, CHD, diabetic, angina and stroke.

Abbreviations: BMI: body mass index; OR: odds ratio; CI: confidence interval. CHD: Coronary heart disease

**Table S2.** Weighted multivariate logistic regression analysis of inflammatory and nutritional indices and cataract after multiple imputation.

| **Variables** | **Model 1** | **Model 2** | **Model 3** |
| --- | --- | --- | --- |
|  | OR (95% CI) *P*-value | OR (95% CI) P-value | OR (95% CI) P-value |
| Ln-NAR | 1.65(1.32, 2.06), <0.001 | 1.85(1.30, 2.61), 0.001 | 1.79(1.22, 2.63), 0.008 |
| Ln-MAR | 3.24(2.64, 3.98), <0.001 | 1.86(1.33, 2.59), <0.001 | 1.81(1.26, 2.60), 0.005 |
| Ln-AISI | 1.71(1,45, 2.02), <0.001 | 1.45(1.19, 1.77), <0.001 | 1.44(1.16, 1.77), 0.004 |
| Ln-ALI | 0.42(0.34, 0.51), <0.001 | 0.69(0.54, 0.89), 0.006 | 0.69(0.52, 0.92), 0.018 |

Model 1: unadjusted

Model 2: Model 1+age, sex and ethnicity

Model 3: Model2 + educational level, marital status, BMI, economic level, smoking status, alcohol consumption, hypertension, CHD, diabetic, angina and stroke.

Abbreviations: BMI: body mass index; OR: odds ratio; CI: confidence interval. CHD: Coronary heart disease

**Table S3.** Comparison between excluded and included participants from NHANES 2005–2008

|  | All | Exclude | Included | *p*-value |
| --- | --- | --- | --- | --- |
| Number | 10905 | 2711 | 8194 |  |
| Gender (N, %) |  |  |  | 0.178 |
| Male | 5289 (48.5) | 1284(47.4) | 4005(48.9) |  |
| Female | 5616 (51.5) | 1427(52.6) | 4189(51.1) |  |
| Age [years, mean (SD)] | 49.66 (18.53) | 51.39 (19.86) | 49.08 (18.04) | <0.001 |
| Ethnicity (N, %) |  |  |  | <0.001 |
| Mexican American | 2034 (18.7) | 525(19.4) | 1509(18.4) |  |
| Other Hispanic | 817 (7.5) | 244(9.0) | 573(7.0) |  |
| Non-Hispanic White | 5254 (48.2) | 1108(40.9) | 4146(50.6) |  |
| Non-Hispanic Black | 2348 (21.5) | 685(25.3) | 1663(20.3) |  |
| Other | 452 (4.1) | 149(5.5) | 303(3.7) |  |
| Education (N, %) |  |  |  | <0.001 |
| Less Than 9th Grade | 1439 (13.2) | 498(18.4) | 941(11.5) |  |
| 9-11th Grade | 1816 (16.7) | 474(17.5) | 1342(16.4) |  |
| Highschool graduate or equivalent | 2646 (24.3) | 673(24.8) | 1973(24.1) |  |
| Some College or AA degree | 2924 (26.8) | 668(24.6) | 2256(27.5) |  |
| College graduate or above | 2080 (19.1) | 398(14.7) | 1682(20.5) |  |
| BMI (N, %) |  |  |  | <0.001 |
| <25 kg/m ^2^ | 3166 (30.5) | 752(34.2) | 2414(29.5) |  |
| 25-30 kg/m ^2^ | 3546 (34.1) | 699(31.8) | 2847(34.7) |  |
| >30 kg/m ^2^ | 3680 (35.4) | 747(34.0) | 2933(35.8) |  |
| Economic level (N, %) |  |  |  | <0.001 |
| <1 | 2122 (19.5) | 607(22.4) | 1515(18.5) |  |
| 1-3 | 4697 (43.1) | 1249(46.1) | 3448(42.1) |  |
| >3 | 4086 (37.5) | 855(31.5) | 3231(39.4) |  |
| Marital status (N, %) |  |  |  | <0.001 |
| Married or living with a partner | 6610 (60.6) | 1474(54.4) | 5136(62.7) |  |
| Unmarried or other | 4295 (39.4) | 1237(45.6) | 3058(37.3) |  |
| Alcohol consumption (N, %) |  |  |  | <0.001 |
| Yes | 7468 (68.5) | 1720(63.4) | 5748(70.1) |  |
| No | 3437 (31.5) | 991(36.6) | 2446(29.9) |  |
| Smoking status (N, %) |  |  |  | 0.029 |
| Never | 5751 (52.7) | 1484(54.7) | 4267(52.1) |  |
| Now | 2413 (22.1) | 592(21.8) | 1821(22.2) |  |
| Former | 2741 (25.1) | 635(23.4) | 2106(25.7) |  |
| Hypertension (N, %) |  |  |  | 0.027 |
| Yes | 3696 (34.0) | 961 (35.7) | 2735 (33.4) |  |
| No | 7188 (66.0) | 1729(64.3) | 5459(66.6) |  |
| Diabetes (N, %) |  |  |  | 0.006 |
| Yes | 1818 (16.7) | 499(18.4) | 1319(16.1) |  |
| No | 9087 (83.3) | 2212(81.6) | 6875(83.9) |  |
| CHD (N, %) |  |  |  | 0.001 |
| Yes | 463 (4.2) | 147(5.4) | 316(3.9) |  |
| No | 10442 (95.8) | 2564(94.6) | 7878(96.1) |  |
| CHF (N, %) |  |  |  | <0.001 |
| Yes | 403 (3.7) | 157(5.8) | 246(3.0) |  |
| No | 10502 (96.3) | 2554(94.2) | 7948(97.0) |  |
| Angina (N, %) |  |  |  | <0.001 |
| Yes | 328 (3.0) | 109(4.0) | 219(2.7) |  |
| No | 10577 (97.0) | 2602(96.0) | 7975(97.3) |  |
| Stroke (N, %) |  |  |  | <0.001 |
| Yes | 452 (4.1) | 158(5.8) | 294(3.6) |  |
| No | 10453 (95.9) | 2553(94.2) | 7900(96.4) |  |

Abbreviations: SD: Standard Deviation; BMI: body mass index; CHD: coronary heart disease.
